# Allelic variation in the canine Cox-2 promoter causes hypermethylation of the canine Cox-2 promoter in clinical cases of renal dysplasia

**DOI:** 10.1186/1868-7083-6-7

**Published:** 2014-04-03

**Authors:** Mary H Whiteley

**Affiliations:** 1 DOGenes Inc., 161 Sherin Ave., Peterborough, ON K9J 7 V5, Canada

**Keywords:** Promoter methylation, Epigenetics, Renal dysplasia, SP1, CpG island, Cyclooxygenase-2

## Abstract

**Background:**

Novel allelic variants in the promoter of the canine cyclooxygenase-2 (Cox-2) gene are associated with renal dysplasia (RD). These variants consist of either deletions of putative SP1 transcription factor-binding sites or insertions of tandem repeats of SP1-binding sites located in the CpG island just upstream of the ATG translation initiation site. The canine Cox-2 gene was studied because Cox-2-deficient mice have renal abnormalities and a pathology that is strikingly similar to RD in dogs.

**Findings:**

The allelic variants were associated with hypermethylation of the Cox-2 promoter only in clinical cases of RD. The wild-type allele was never methylated, even in clinical cases that were heterozygous for a mutant allele. In cases that were biopsy-negative, the promoter remained unmethylated, regardless of the genotype. Methylated DNA was found in DNA from various adult tissues of dogs with clinical RD.

**Conclusions:**

The mechanism of action of the allelic variation in the canine Cox-2 promoter most likely involves variation in the extent of epigenetic downregulation of this gene. This epigenetic downregulation must have occurred early in development because methylated Cox-2 promoter DNA sequences are found in various adult tissues.

## Findings

Renal dysplasia (RD) or improper development of the kidneys is a life-threatening disease that can result in end-stage renal failure. In all species, RD can be genetic or acquired. There has been intense speculation regarding the role of epigenetic modifications in kidney development [1]; however, to my knowledge this report is the first documented case involving allele-specific epigenetic changes associated with kidney disease.

Cyclooxygenase-2 (Cox-2) is generally considered to be a rate-limiting enzyme for prostaglandin synthesis in response to biological events such as injury, inflammation, and proliferation [2]. Transcriptional regulation of Cox-2 is complex. In most tissues, Cox-2 requires activation by inflammatory or other stimuli and is regulated by numerous 5′ transcription factors in addition to post-transcriptional regulation through elements at the 3′ untranslated region of the gene [3]. However, in the adult kidney and several other organs, Cox-2 is constitutively expressed in various cell types, and its expression increases under non-physiological conditions [4]. Furthermore, Cox-2 also functions as a developmental gene, as evidenced by knockout and knockdown mouse models that show abnormal kidney development [5-7]. Cox-2 knockdown mice have a less severe phenotype than Cox-2 knockout strains. Furthermore, expression studies in developing rats show that Cox-2 has highly specific and tightly regulated gene expression.

In the developing rat Cox-2 protein and mRNA are not detected until mid-gestation, but Cox-2 expression is high from day 15 and increases up to day 20. In the fetus, Cox-2 expression is restricted to the skin, heart, cartilage, and kidney [8]. A similar pattern of Cox-2 expression has been reported during mouse and human development [9,10].

Cox-2-deficient mice have renal abnormalities and a pathology that is very similar to RD in dogs [5,6]. DNA sequencing of the canine Cox-2 gene revealed allelic variants with insertions or deletions of SP1-binding sites just upstream of the ATG translation start site [11].

**Figure 1:**
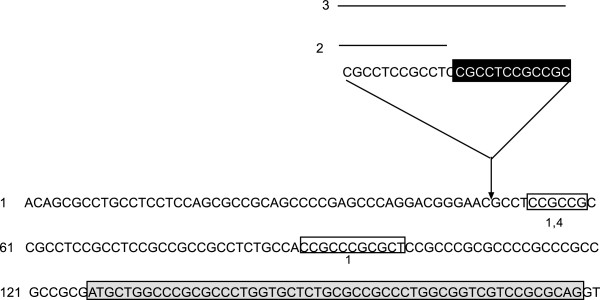
**Allelic variants in the cyclooxygenase-2 (Cox-2) promoter, just upstream of the ATG translation start site.** Allelic variant 1 has DNA sequences that are deleted; these sequences are boxed with the number 1 below the box. Allelic variants 2 and 3 contain insertions. Allele 2 is underlined and not shaded. Allele 3 contains a further insertion of sequences at nucleotide -78 relative to the ATG start, and is a duplication of the inserted sequences in allele 2. Allelic variant 4 contains a deletion of 6 nucleotides (CCGCCG) and is boxed and marked with the number 4 below the box. The sequences of the complete promoter region and 5′ untranslated region are deposited in GenBank under accession number AY927786.1.

There are four allelic variants of the wild-type allele, and sequence numbering in all alleles is relative to the translation start codon. Allele 1 contains a 6-nucleotide deletion (CCGCCG) at position -73 of the canine Cox-2 gene and a deletion of 11 nucleotides at position -37 (CCGCCCGCGCT). Allele 2 contains an insertion of 12 nucleotides (CGCCTCCGCCTC) starting at position -78. Allele 3 contains an insertion of 24 nucleotides (CGCCTCCGCCTCCGCCTCCGCCGC) at position -78. The first 12 nucleotides of this 24-nucleotide insertion comprise the insertion in allele 2. Allele 4 comprises the 6-nucleotide deletion at position -73 found in allele 1 (Figure 1). The frequency of the mutant alleles varies among different breeds, as shown in a previous study [11].

These allelic variants exist within a large CpG island in the Cox-2 promoter near the translation start site; this CpG island is approximately 450 nucleotides long (as predicted by MethPrimer) [12]. Normally, the CpG island in the human Cox-2 promoter is unmethylated. However, hypermethylation of the human Cox-2 CpG island has been identified in some types of cancer [13,14], in which the level of Cox-2 repression increased with higher levels of methylation.

The aim of this study was to determine whether these allelic variants cause hypermethylation of the CpG island in the canine Cox-2 promoter in clinical cases of RD. Such hypermethylation would provide a mechanism by which these alleles could decrease or silence COX-2 expression during a critical time of development and generate a phenotype that is similar to the mouse knockout [5,6] and knockdown [7] models of RD.

### DNA samples used in the study

DNA samples were obtained from cheek cells or preserved kidney tissue (preserved in RNALater (Qiagen Inc., Valencia, CA, USA) or as formalin-fixed and paraffin-embedded tissue sections). All of the samples were harvested in a previous study [11]. For bisulfite conversion, the DNA from the cheek swabs was concentrated using a DNA Clean & Concentrator™ kit from Zymo Research Corp. (Irvine, CA, USA). The DNA from kidney tissue preserved in RNALater was extracted using a mini gDNA extraction kit (Zymo Research Corp.).

DNA samples were obtained from five dogs with clinical cases of moderate to severe RD (>35% fetal glomeruli on biopsy). In addition, there were three biopsy-negative samples and five samples from randomly chosen dogs with a homozygous wild-type Cox-2 genotype. A negative biopsy indicated that no fetal glomeruli were present in the kidney section examined.

### Bisulfite conversion and methylation-specific PCR

The DNA was bisulfite modified using the EZ DNA Methylation-Lightning™ Kit (Zymo Research Corp.). Primers for methylation-specific PCR [15] and DNA sequencing were chosen using MethPrimer [12] as follows.

Methylated DNA-specific primer set:

M forward primer, 5′-TGTTTTTTTTAGCGTCGTAGTTTC-3′

M reverse primer, 5′-AAAAACCAAATACCCACCTACG-3′

Unmethylated DNA primer set:

Forward primer, 5′-TGTTTTTTTTAGTGTTGTAGTTTTGA-3′

Reverse primer, 5′-AAAAACCAAATACCCACCTACAC-3′

The PCR conditions were 95°C for 1 min, followed by 35 cycles of 95°C for 15 s, 52°C for 15 s, and 72°C for 10 s, using MyTaq hot-start polymerase (Bioline, Taunton, MA, USA).

To obtain sufficient DNA for sequencing of the methylated DNA, the methylation-specific PCR products were separated on a PCR-CheckIT gel (Elchrom Scientific AG, Cham, Switzerland) with 1× buffer as recommended by the manufacturer. Individual alleles were recovered from the gel using BandPick™ (Elchrom Scientific) and re-amplified with the primers described above for DNA sequencing. The DNA was re-amplified using MyTaq hot-start polymerase for 30 cycles. The fragments were sequenced by Macrogen Corp. (Rockville MD, USA).

The PCR products from methylation-specific PCR were separated on a Spreadex 300 gel (Elchrom Scientific) with 1× TAE as the running buffer. Xylene cyanol loading buffer was run to the bottom of the gel.

## Results

**Figure 2:**
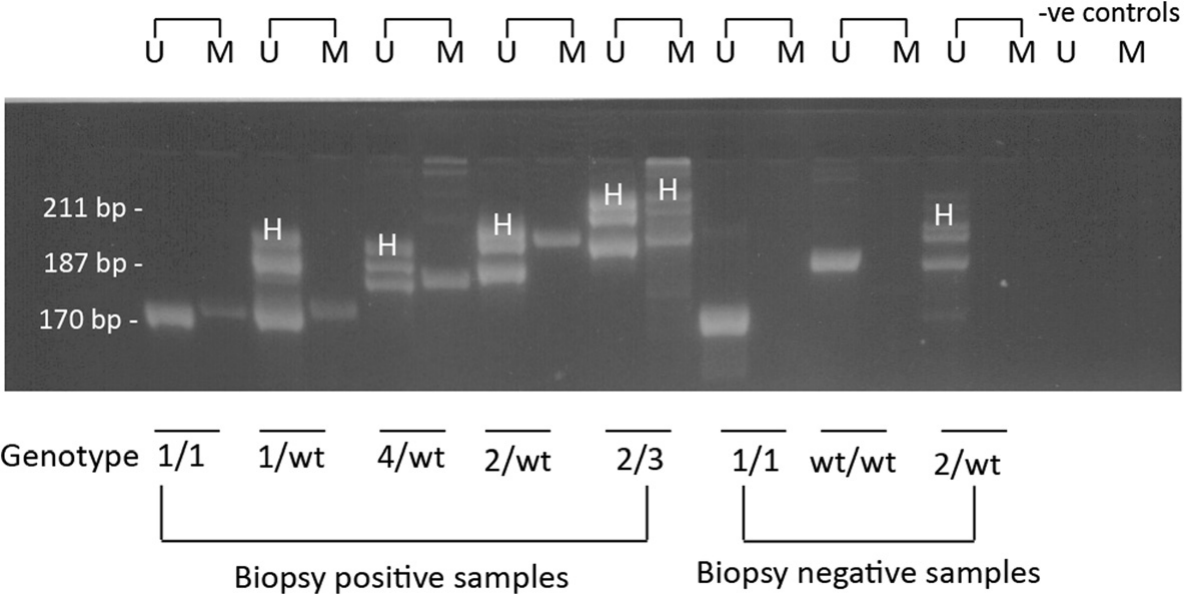
**Representative results of methylation-specific PCR experiments with the dogs described in Table**1**.** The sizes of the alleles are as follows: wild-type allele, 187 bp; Allele 1, 170 bp; Allele 2, 99 bp; Allele 3, 211 bp; Allele 4, 181 bp. Cases 1–5 are biopsy-positive dogs. Cases 6–8 are biopsy-negative dogs. U is the unmethylated DNA from the methylation-specific PCR, and M is the methylated DNA. The final two lanes represent negative controls (no DNA) for the methylation-specific PCR experiments. In the case of heterozygotes, a heteroduplex band (marked with an H) is presented above the actual alleles. wt, Wild-type.

The results of the methylation-specific PCR experiments are summarized in Figure 2 and Table 1. The CpG island of the canine Cox-2 wild-type allele was always unmethylated. Methylated DNA was only found in RD dogs that were either homozygous or heterozygous for a mutant allelic variant. In RD heterozygotes, only the mutant allele was methylated. In the case of RD dogs that were homozygous for mutant alleles, both alleles were methylated. In biopsy-negative dogs that were homozygous wild type, heterozygous, or homozygous for mutant alleles, the Cox-2 promoter remained unmethylated (Figure 2 and Table 1).

**Table 1:** Genotypes and clinical information for dogs used in this study

**Case number**	**Breed**	**Biopsy**	**Genotype**	**Methylated DNA products**	**Source of DNA**	**Age at death from renal failure**
1	Cocker spaniel	+	Allele1/Allele1	Allele1	Formalin preserved kidney tissue	2.5 years
2	Shih Tzu	+	Allele1/wt	Allele1	Cheek cells	4 months
3	Standard poodle	+	Allele2/wt	Allele2	Kidney	9 months
4	Lhasa apso	+	Allele2/Allele3	Allele2/Allele3	Kidney	8 years
5	Gordon Setter	+	Allele4/wt	Allele4	Cheek cells	3.5 years
6	Lhasa apso	-	wt/wt	None	Cheek cells	n/a
7	Miniature Schnauzer	-	Allele1/Allele1	None	Kidney	n/a
8	Lhasa apso	-	Allele2/Allele2	None	Cheek cells	n/a
9	English cocker spaniel	n/a	wt/wt	None	Cheek cells	n/a
10	Boxer	n/a	wt/wt	None	Cheek cells	n/a
11	Collie	n/a	wt/wt	None	Cheek cells	n/a
12	Collie	n/a	wt/wt	None	Cheek cells	n/a
13	Cocker spaniel	n/a	wt/wt	None	Cheek cells	n/a

Allele1–allele 4 are the variants shown in Figure 1. +, Indicates that biopsy data showed moderate to severe renal dysplasia (>35% fetal glomeruli); –, indicates that no fetal glomeruli were found in the biopsy; n/a, not available; wt, wild-type.

**Figure 3:**
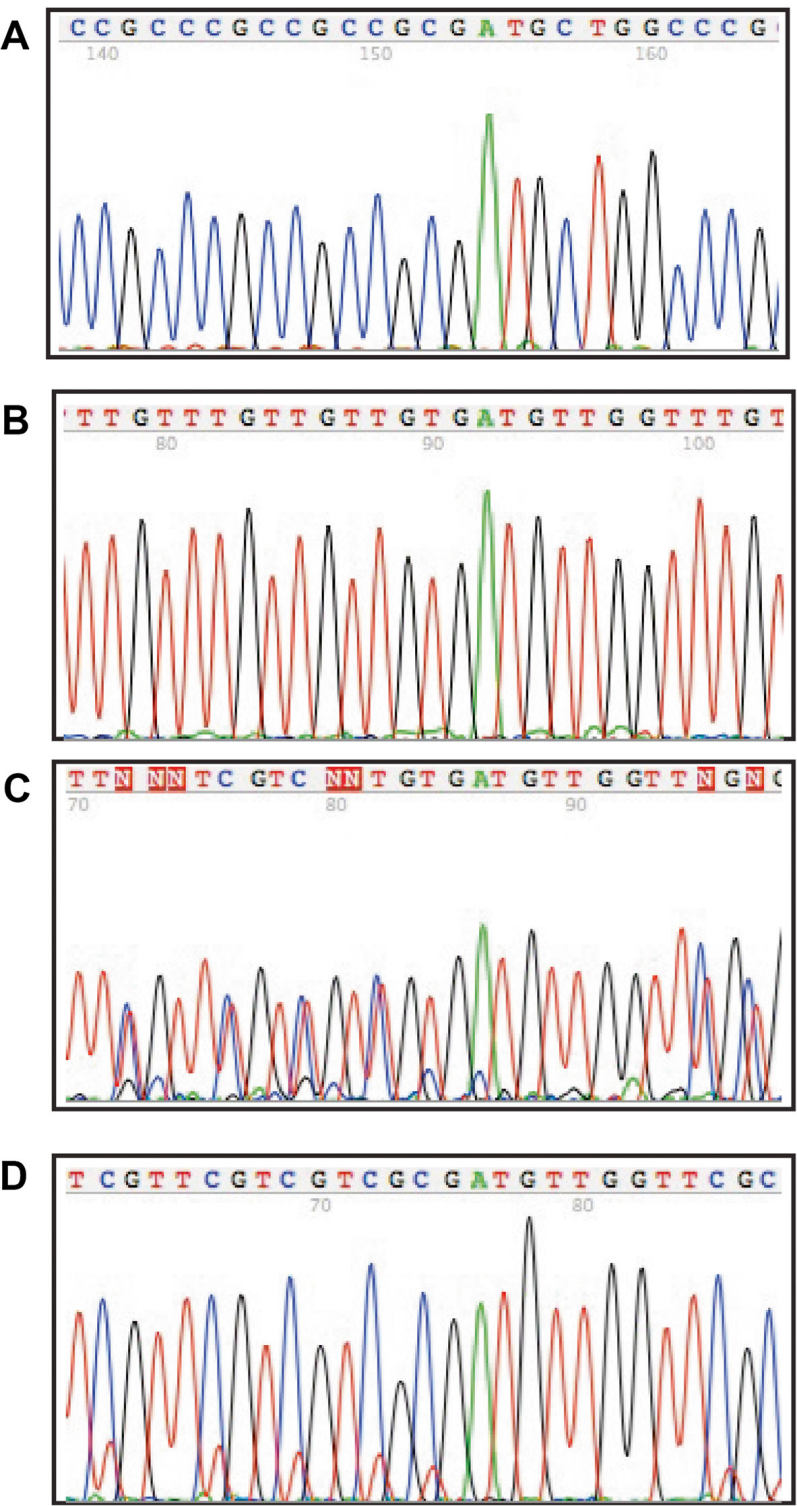
**Representative electropherograms from the DNA sequencing of bisulfite-converted DNA. (A)** DNA that has not been converted with bisulfite (normal sequence). The primer set for this experiment was primer set 1 from reference [11]. **(B)** Wild-type allele showing that the CpG island of the Cox-2 promoter is unmethylated. **(C)** Electropherogram showing dense methylation of multiple alleles throughout the CpG island of the cyclooxygenase-2 (Cox-2) gene. **(D)** Electropherogram from Case number 2 (Shiz tzu) showing complete hypermethylation of the Cox-2 CpG island.

The methylation-specific PCR experiments were validated by DNA sequencing. DNA sequencing of methylated DNA from clinical samples generally showed dense methylation of multiple alleles. However, in case number 2 (Table 1), the Shih tzu (Allele 1/wild type) allele 1 was hypermethylated at all CG dinucleotide positions throughout the amplified product (Figure 3).

## Discussion

The first evidence that Cox-2 acts a critical gene in development was obtained from Cox-2 knockout mouse strains, which show severe defects in their kidneys [5,6]. Further studies revealed a specific and tightly regulated pattern of gene expression in developing rats, mice, and humans [8-10]. The defects noted in the kidney included immature glomeruli, and the severity of RD in dogs is classified by the percentage of fetal glomeruli found on biopsy.

This study shows that novel allelic variants in the CpG island of the Cox-2 promoter have aberrant methylation of the Cox-2 promoter only in clinical cases of RD. The wild-type alleles are never methylated, even in animals that are heterozygous for a mutant allele. This in effect provides an internal control for methylation and shows a distinct resistance of the wild-type chromosome to methylation. This finding of methylation resistance was further reinforced by the lack of methylation in homozygous wild-type controls. These results provide strong evidence that the mechanism of action of the mutant alleles likely involves some degree of transcriptional downregulation via methylation of the CpG island in the Cox-2 promoter. Such a mechanism is supported by a previous study on cancer cell lines, in which methylation of the Cox-2 promoter resulted in decreased expression or silencing of the Cox-2 gene, depending on the extent of methylation [13,14]. These findings are consistent in different types of cancer. The results of the present study also explain the mode of inheritance as dominant with incomplete penetrance because the degree of methylation varies between the clinical cases. The occurrence of aberrant methylation in different breeds and therefore in different genetic backgrounds reinforces the notion of a universal mechanism of action.

In general, Cox-2 is an inducible enzyme, but it has a basal level of expression in a subset of adult kidney tissues that is elevated in response to external stimuli, highlighting the complex regulation of this gene [3]. In a previous study, RT-PCR showed Cox-2 expression in adult kidneys, although this expression could have been in response to the inflammation intrinsic to this disease, and the expression may be regulated by transcription factors other than tissue-specific or developmental transcription factors [11]. Although Cox-2 is expressed in a limited number of fetal tissues, the only defects observed in Cox-2 knockout and knockdown mouse models are dysplastic kidneys, suggesting that downregulation of Cox-2 may be tissue-specific in the developing fetus [5-7]. Embryogenesis would therefore be the critical time to evaluate downregulation of Cox-2; however, observation during embryogenesis is not practical in dogs.

For the proposed mechanism of action to work, methylated DNA must be present in kidney tissues. However, methylated DNA was also found in cheek cells, indicating that the epigenetic event occurred early in development and in cells of both mesodermal and ectodermal origin. The methylation may thus have been caused by either environmental factors or other genes.

There has been intense speculation regarding the role of epigenetics in kidney disease, and this study represents the first example of DNA methylation of a CpG island that is likely to be involved in a developmental disease process. Other genetically inherited dominant diseases with incomplete penetrance may involve a similar mechanism.

## Abbreviations

Cox-2: cyclooxygenase-2; PCR: polymerase chain reaction; RD: renal dysplasia; RT-PCR: reverse transcription PCR.

## Competing interests

Patent pending: Inventor: WHITELEY, Mary, Helen; (CA). (WO/2009/092171) COMPOSITIONS AND METHODS FOR DETECTING JUVENILE RENAL DYSPLASIA OR CALCIUM OXALATE STONES IN DOGS. Filed on January 2009. MW owns 10 common shares in DOGenes, Inc. and is employed by DOGenes, Inc. The funding for this research, including the cost of publication, was a capital expense of DOGenes.
